# CD54^+^ rabbit adipose-derived stem cells overexpressing HIF-1α facilitate vascularized fat flap regeneration

**DOI:** 10.18632/oncotarget.16777

**Published:** 2017-04-01

**Authors:** De-Quan Li, Guan-Ming Lu, Yi-Dan Liang, Zhi-Jie Liang, Min-Hong Huang, Qi-Liu Peng, Dong-Hua Zou, Rong-He Gu, Fang-Tian Xu, Hui Gao, Zhen-Dong Chen, Guang-Yi Chi, Zhong-Heng Wei, Li Chen, Hong-Mian Li

**Affiliations:** ^1^ Department of Mammary Glands Surgery, The Third Hospital of Nanchang City, Nanchang 330009, China; ^2^ Department of Glands Surgery, The Affiliated Hospital of Youjiang Medical University for Nationalities, Baise 533000, China; ^3^ Central Laboratory of Medical Science, The Fifth Affiliated Hospital of Guangxi Medical University, Nanning 530022, China; ^4^ Department of Mammary Glands Surgery, The Fifth Affiliated Hospital of Guangxi Medical University, Nanning 530022, China; ^5^ Department of Neurology, The Fifth Affiliated Hospital of Guangxi Medical University, Nanning 530022, China; ^6^ Department of Orthopedic Surgery, The Fifth Affiliated Hospital of Guangxi Medical University, Nanning 530022, China; ^7^ Department of Orthopedics, The First Affiliated Hospital of Gannan Medical University, Ganzhou 341000, China; ^8^ Department of Plastic and Aesthetic Surgery, The Fifth Affiliated Hospital of Guangxi Medical University, Nanning 530022, China; ^9^ Department of Neurology, The First Affiliated Hospital of Guangxi Medical University, Nanning 530021, China

**Keywords:** rabbit adipose-derived stem cells, HIF-1α, adipogenic differentiation, engineered fat flaps, angiogenesis

## Abstract

Fat flap transplantation is frequently performed in patients suffering from soft tissue defects resulting from disease or trauma. This study explored the feasibility of constructing vascularized fat flaps using rabbit adipose-derived stem cells (rASCs) and collagen scaffolds in a rabbit model. We evaluated rASCs proliferation, paracrine function, adipogenesis, vascularization, and CD54 expression, with or without HIF-1α transfection *in vitro* and *in vivo*. We observed that adipogenic differentiation potential was greater in rASCs with high CD54 expression (CD54^+^rASCs) than in those with low expression (CD54^–^rASCs), both *in vitro* and *in vivo*. HIF-1α overexpression not only augmented this effect, but also enhanced cell proliferation and paracrine function *in vitro*. We also demonstrated that HIF-1α-transfected CD54^+^rASCs showed enhanced paracrine function and adipogenic capacity, and that paracrine function increases expression of angiogenesis-related markers. Thus, CD54^+^rASCs overexpressing HIF-1α enhanced large volume vascularized fat flap regeneration in rabbits, suggesting CD54 may be an ideal candidate marker for ASCs adipogenic differentiation.

## INTRODUCTION

Soft tissue regeneration via adipose tissue engineering is a growing field addressing current clinical demands related to various soft tissue defects [1–4]. Soft tissue substitutes are often required following disease or tissue trauma, such as in adipose or muscle tissues. Soft tissue defects may affect patient physical appearance, cause emotional disturbances, and/or impair function. Currently, autologous tissue flaps, fat, or commercially available fillers are commonly used in reconstructive surgeries, specifically for repairing facial tissue defects and tumor resection sites. Although these strategies achieve clinical success, subsequent volume losses and secondary donor-site deformities challenge long-term efficacies. Emerging tissue engineering strategies represent innovative approaches for many clinical challenges. Such strategies include incorporating seed cells, biodegradable scaffolds, and microenvironments to provide specific inductive signals for tissue regeneration.

Adipose-derived stem cells (ASCs) represent a readily available supply of mesenchymal stem cells (MSCs) from lipo-aspirates [5–7]. ASCs exhibit enhanced proliferation and reduced senescence, and do not trigger immunological rejection [8]. ASCs may differentiate into chondrocytes, osteoblasts, adipocytes, myocytes, or neuronal-phenotype cells *in vitro* or *in vivo* [9–13]. Efforts directed toward promoting ASCs adipogenic differentiation have included growth factor or induction agent administration, other angiogenic methods, and the use of appropriate scaffolds [14–19]. ASCs exhibit poor adipogenic differentiation capacity under non-adipogenic-inducing conditions. We hypothesized that some cluster of differentiation (CD) markers could be closely related to adipogenic differentiation-related functions in ASCs. Our previous work showed that transfecting human breast adipose-derived stem cells (HBASCs) with *CXCR4* effectively improved survival of free fat transplants [20]. We also found that ginsenoside Rg1 and platelet-rich fibrin co-administration improved HBASCs function in soft tissue regeneration engineering [21]. In this study, we purified CD54^+^rASCs using immunomagnetic beads, transfected them with *HIF-1α*, and seeded them on three-dimensional, porous, collagen type I sponge scaffolds *in vitro*. Paracrine function was assessed prior to implantation. Finally, we assessed vascularized fat flaps constructed using *HIF-1α*-transfected CD54^+^rASCs for optimizing adipose tissue regeneration *in vivo*.

## RESULTS

### rASCs growth characterization, immunophenotype, and multipotency

**Figure 1:**
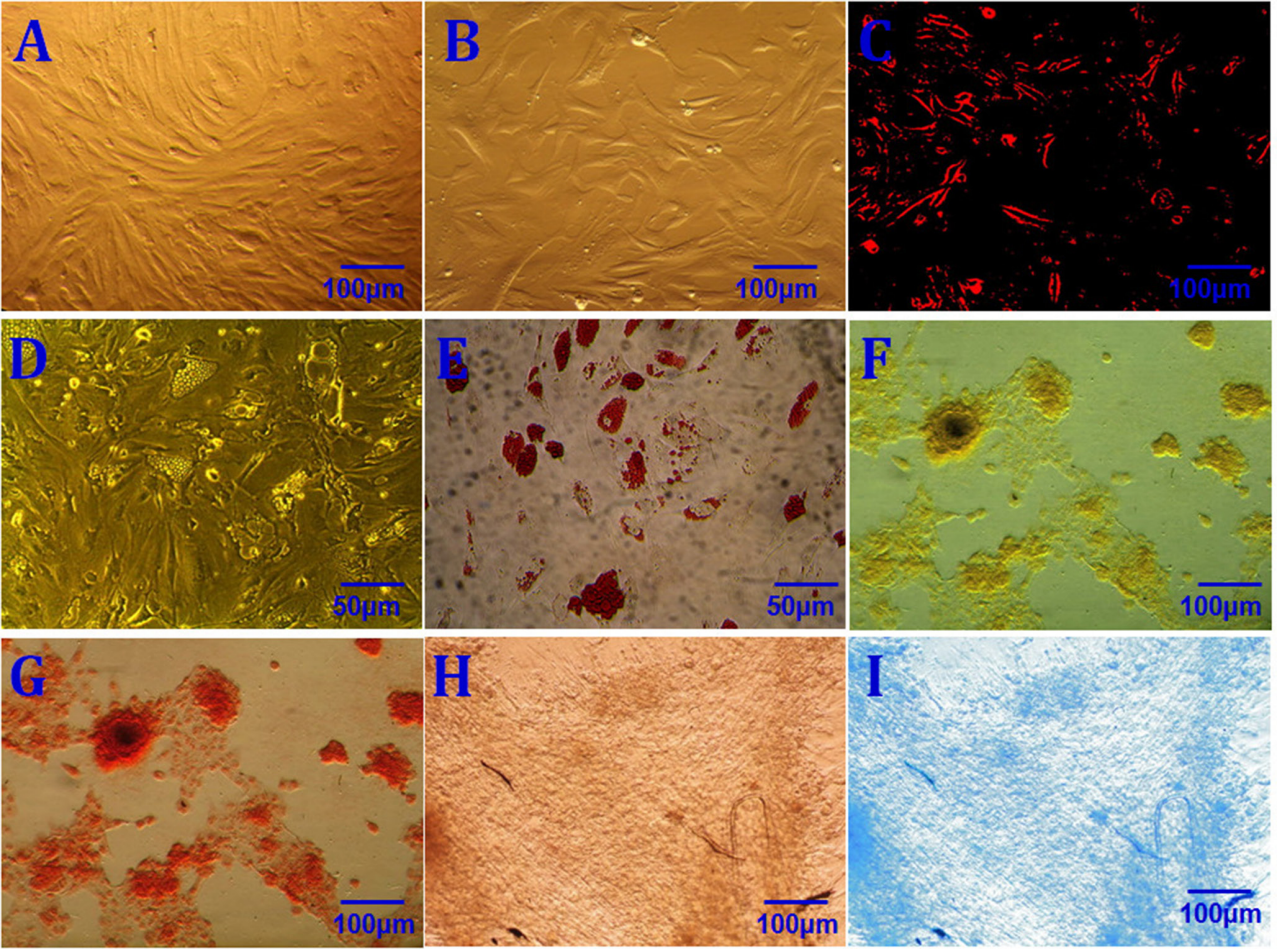
rASCs characterization Morphological characterization of primary rASCs (**A**) Morphological characterization of P3 rASCs (**B**) DiI labeling of rASCs (DiI+) (**C**) Adipogenic induction conducted for 2 weeks before staining (**D**) Positive oil red O staining following 2 weeks of adipogenic induction (**E**) Osteogenic induction conducted for 3 weeks before staining (**F**) Positive alizarin red staining following 3 weeks of osteogenic induction (**G**) Chondrogenic induction conducted for 3 weeks before staining (**H**) Positive alcian blue staining following 2 weeks of chondrogenic induction (**I**) Scale bars: 50 μm (D, E); 100 μm (A–C, F–I).

Homogeneous rASCs growing in a monolayer with a spindle-shaped morphology were observed by phase-contrast microscopy after primary culture for 7–10 d, reaching 80–90% confluence (Figure 1). These rASCs proliferated rapidly. During subculture, P3 rASCs reached the same level of confluence within 3–4 d with a 1:3 split ratio (Figure 1). This demonstrated that rASCs resemble other ASCs in terms of morphology and proliferative capacity. rASCs expressed the mesenchymal surface markers CD29, CD44, CD54, CD73, CD90, CD105, and HLA-ABC, as determined by flow cytometry (Table 1). In contrast, < 7% of rASCs expressed the hematopoietic markers CD31, CD34, and CD45, and the histocompatibility antigen class II HLA-DR-DP-DQ. After DiI labeling, rASCs cytoplasms showed red fluorescence (Figure 1).

**Table 1:** Immunophenotype analysis of passage 3 rASCs

Surface Markers	rASCs at P3 (mean ± SD,%)
CD29	97.3 ± 1.2
CD44	98.1 ± 0.7
CD54	92.4 ± 1.1
CD73	95.2 ± 0.8
CD90	99.2 ± 0.5
CD105	99.4 ± 0.3
HLA-ABC	98.9 ± 0.9
CD31	6.1 ± 0.7
CD34	4.8 ± 0.6
CD45	4.3 ± 0.9
HLA-DP, DQ, DR	3.9 ± 0.5

*n* = 6.

Subconfluent P3 rASCs were cultured with adipogenic, osteogenic, and chondrogenic induction media, and lineage-specific cell morphologies were observed following 2, 3, and 2 weeks of culture, respectively. Adipocytes, osteoblasts, and chondrocytes can be identified by positive oil red O, alizarin red, or alcian blue staining, respectively. Lineage-specific histological staining was performed using these dyes, and results confirmed that rASCs differentiated into adipocytes, osteoblasts, and chondrocytes following culture in the relevant induction medium (Figure 1). These results validated rASCs multipotency

### Effect of HIF-1α transfection on rASCs proliferation *in vitro*

**Figure 2:**
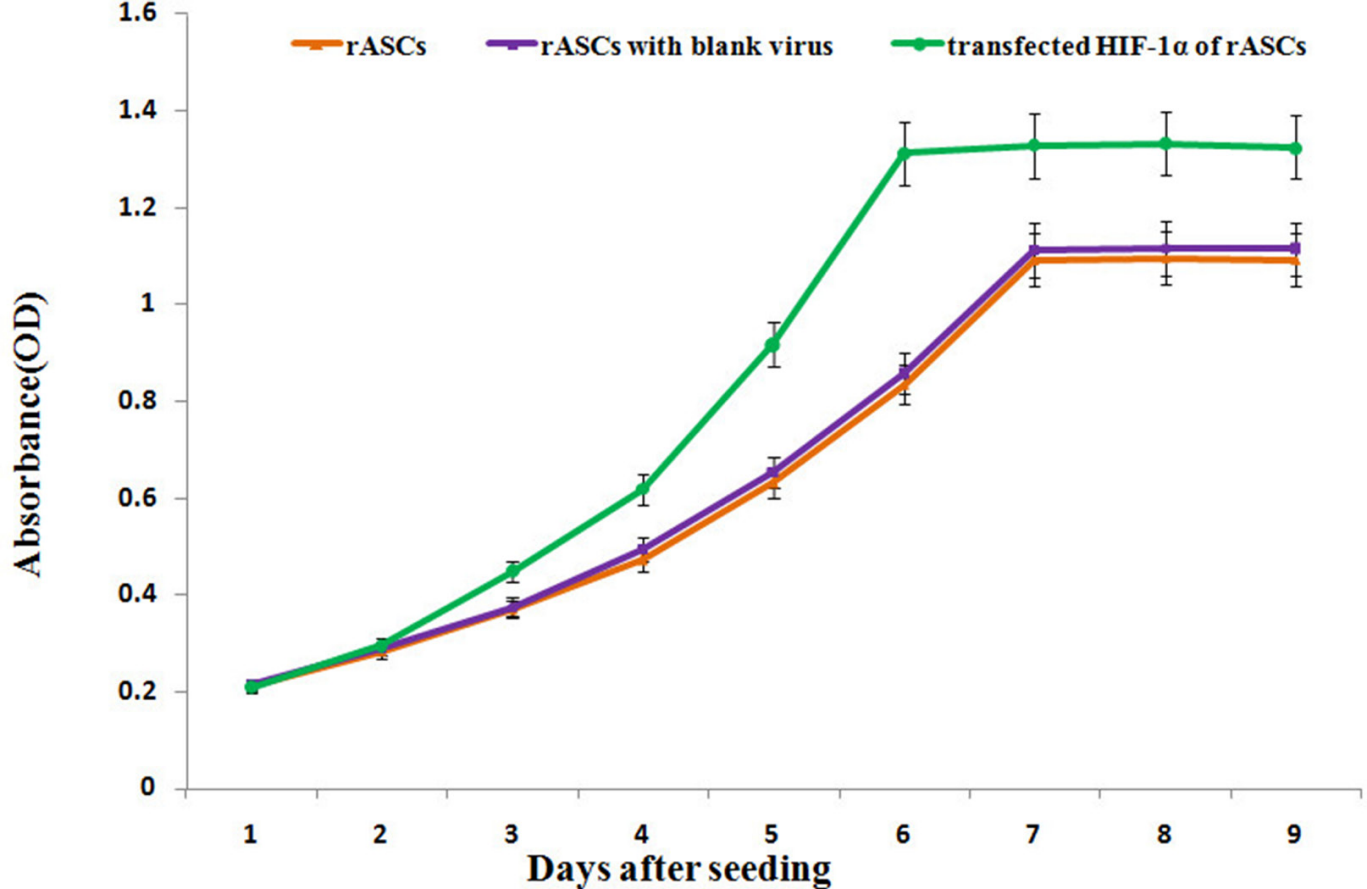
rASCs proliferation as assessed by a CCK-8 assay HIF-1α-transfected rASCs displayed higher absorbance values than blank-transfected and control rASCs at every time point starting from d 3. *n* = 6, *P <* 0.01 (ANOVA).

CCK-8 assays were performed on HIF-1α-transfected, blank virus-transfected, and control rASCs. Beginning 3 d after culture, HIF-1α-transfected cell proliferation rate was higher than that of the blank virus-transfected and control cells, and HIF-1α-transfected cell doubling time was shorter than that of the blank virus -transfected and control cells (*P <* 0.001, Figure 2).

### Paracrine signal levels after rASCs HIF-1α transfection

**Figure 3:**
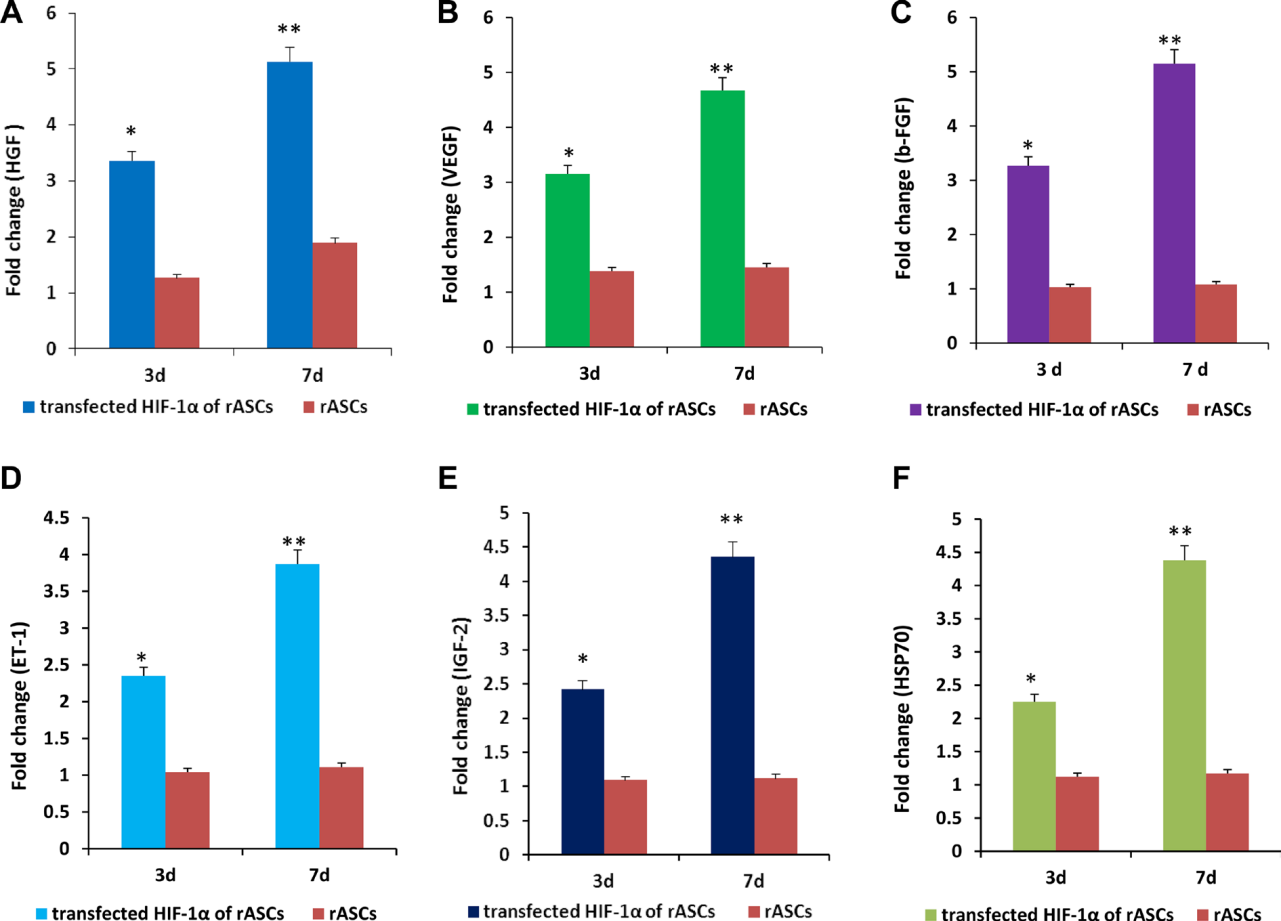
rASCs expression of paracrine factors after HIF-1α transfection *in vitro* Higher HGF (**A**), VEGF (**B**), b-FGF (**C**), ET-1 (**D**), IGF-2 (**E**), and HSP70 (**F**) expression were measured in the treated group than in the control group at 3 and 7 d. *n* = 3, *P <* 0.01 (*t-test*).

Three and 7 d after HIF-1α transfection, supernatant levels of hepatocyte growth factor (HGF), vascular endothelial growth factor (VEGF), endothelin-1 (ET-1), basic fibroblast growth factor (b-FGF), insulin-like growth factor-2 (IGF-2) and heat shock protein 70 (HSP70) were measured in the treatment and control groups by ELISA. After 3 d, supernatant levels of HGF, VEGF, ET-1, b-FGF, IGF-2, and HSP70 were lower in the untransfected group than in the transfected group, with fold changes of 1.27 vs. 3.66, 1.38 vs. 3.15, 1.04 vs. 2.35, 1.03 vs. 3.27, 1.09 vs. 2.42, and 1.12 vs. 2.25, respectively. After 7 d, supernatant levels of HGF, VEGF, ET-1, b-FGF, IGF-2, and HSP70 were also lower in the untransfected group than in the transfected group, with fold changes of 1.89 vs. 5.13, 1.45 vs. 4.67, 1.11 vs. 3.67, 1.08 vs. 5.15, 1.12 vs. 4.36, and 1.17 vs. 4.38, respectively (Figure 3).

### Effect of HIF-1α on rASCs adipogenic differentiation *in vitro*

**Figure 4:**
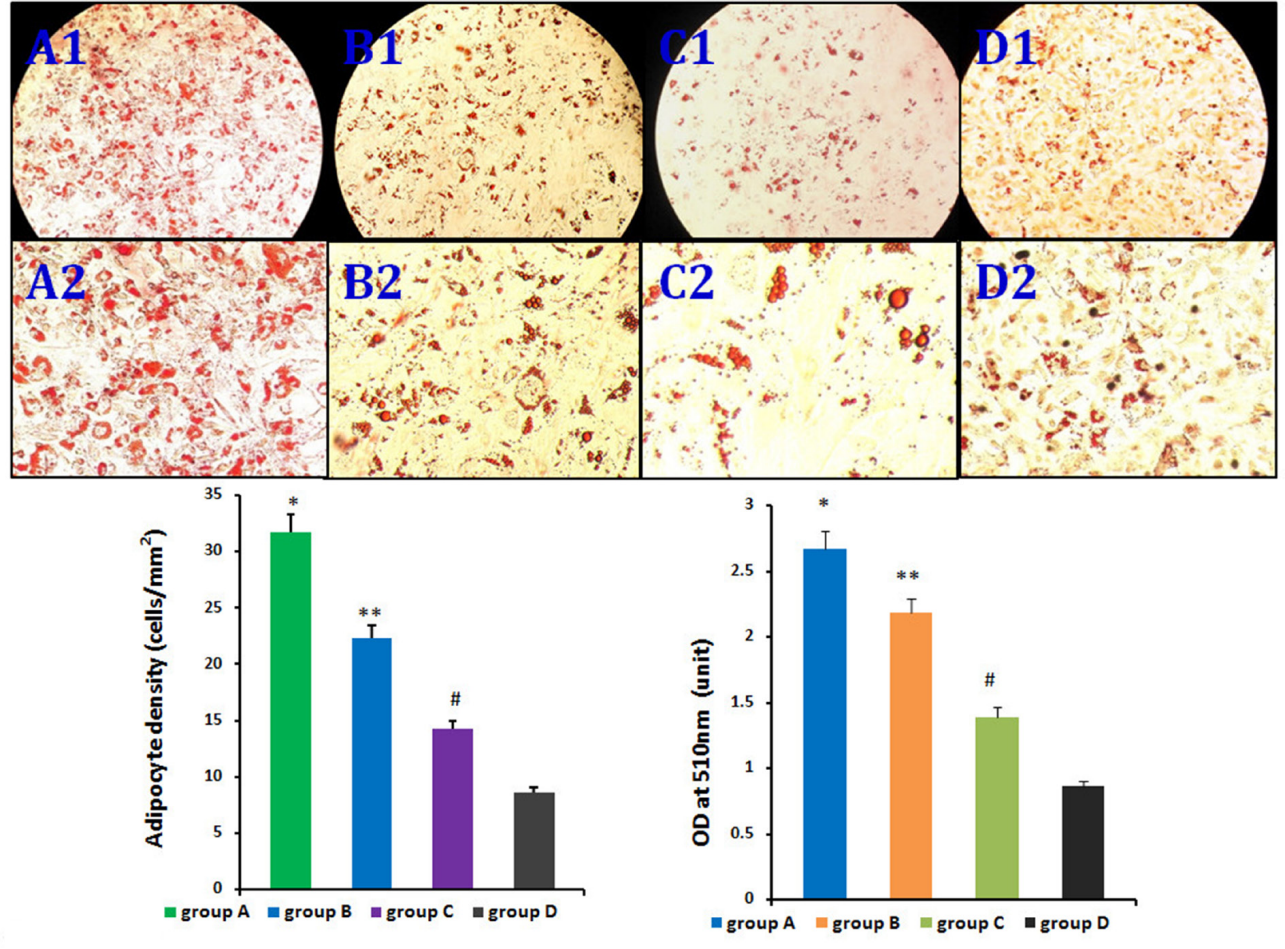
Influence of HIF-1α on rASCs adipogenic differentiation *in vitro* There were differences between group A and B, group A and C, group A and D, groups B and C, groups B and D, and groups C and D in adipocyte density and lipid concentration. *P < 0.01, **P < 0.01, ^#^*P <* 0.01.

The four rASCs groups exhibited different adipogenic potentials *in vitro*. CD54^+^rASCs contained more large oil red O-positive lipid droplets in their cytoplasm, and HIF-1α transfection augmented this effect. However, CD54^-^rASCs transfected with HIF-1α (group C) and untransfected CD54^-^rASCs contained fewer oil red O-positive lipid droplets (Figure 4). There were differences between groups A and B, groups A and C, groups A and D, groups B and C, groups B and D, and groups C and D in adipocyte density and lipid concentration (Figure 4).

### Neogenetic fat flaps macroscopic findings and histopathological assessment

**Figure 5:**
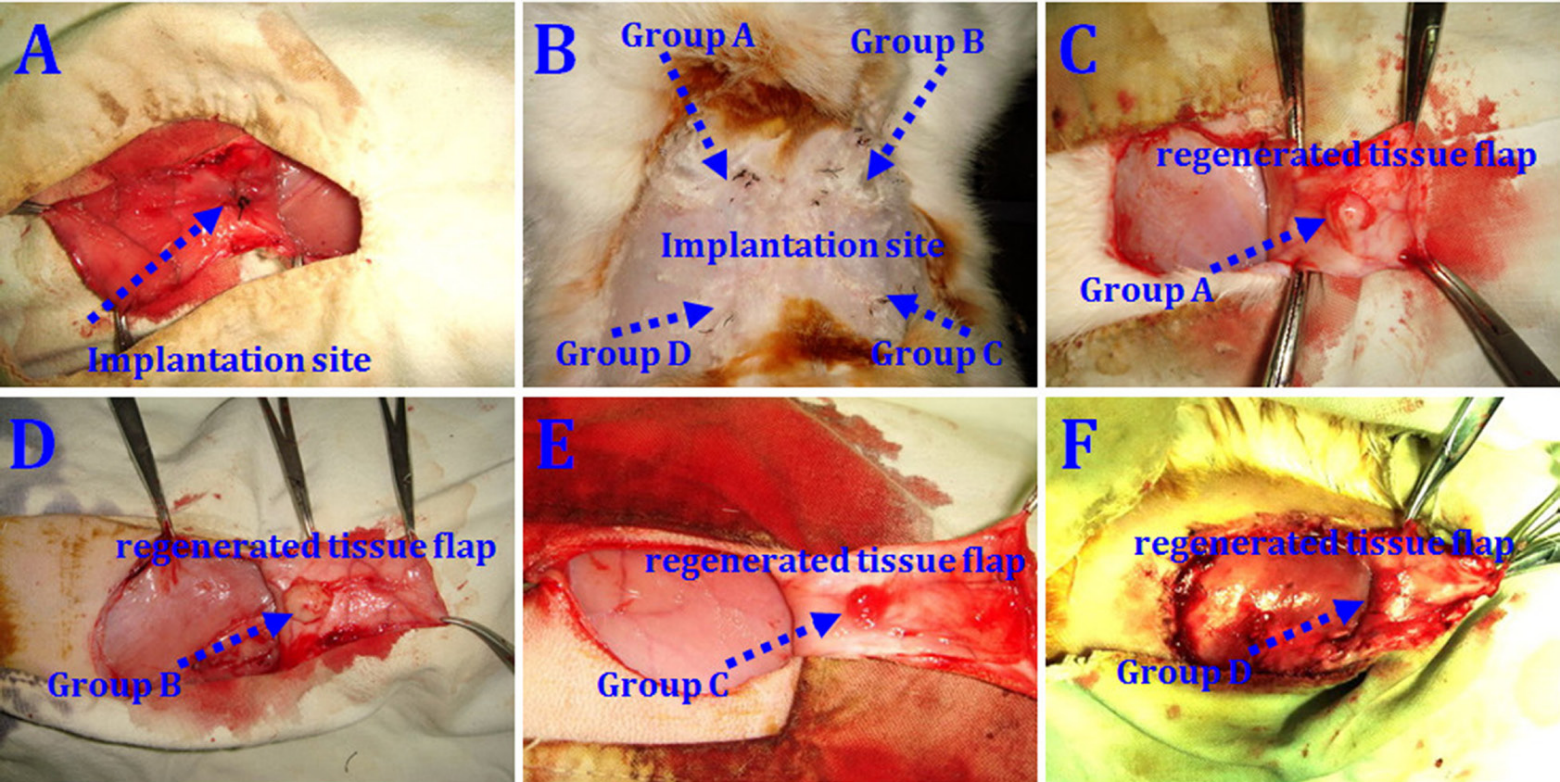
rASCs-loaded scaffold implantation *in vivo* Cell-loaded scaffolds implanted immediately after the operation (**A**) At 12 weeks post-implantation, collagen scaffolds loaded with HIF-1α-transfected CD54^+^rASCs (group A), CD54^+^rASCs (group B), HIF-1α-transfected CD54^-^rASCs (group C), and CD54^-^rASCs (group D) (**B**) Macroscopic findings of neogenetic tissue in groups A (**C**), B (**D**), C (**E**), and D (**F**).

**Figure 6:**
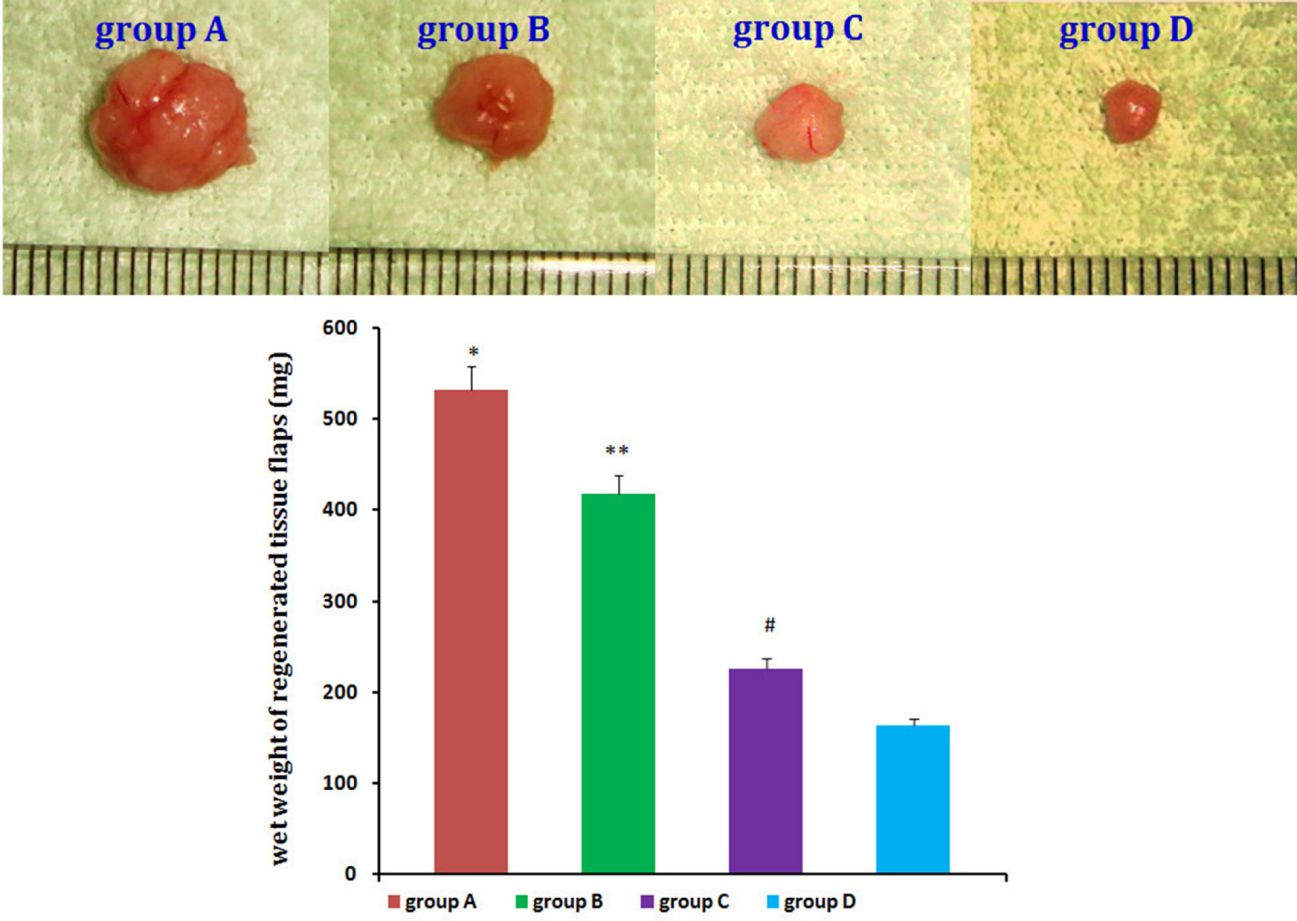
Neogenetic fat flap macroscopic findings Wet weights of the neogenetic fat flaps in the four groups were measured and compared. *n* = 20, *P < 0.01, **P < 0.01, ^#^*P <* 0.01 (ANOVA).

**Figure 7:**
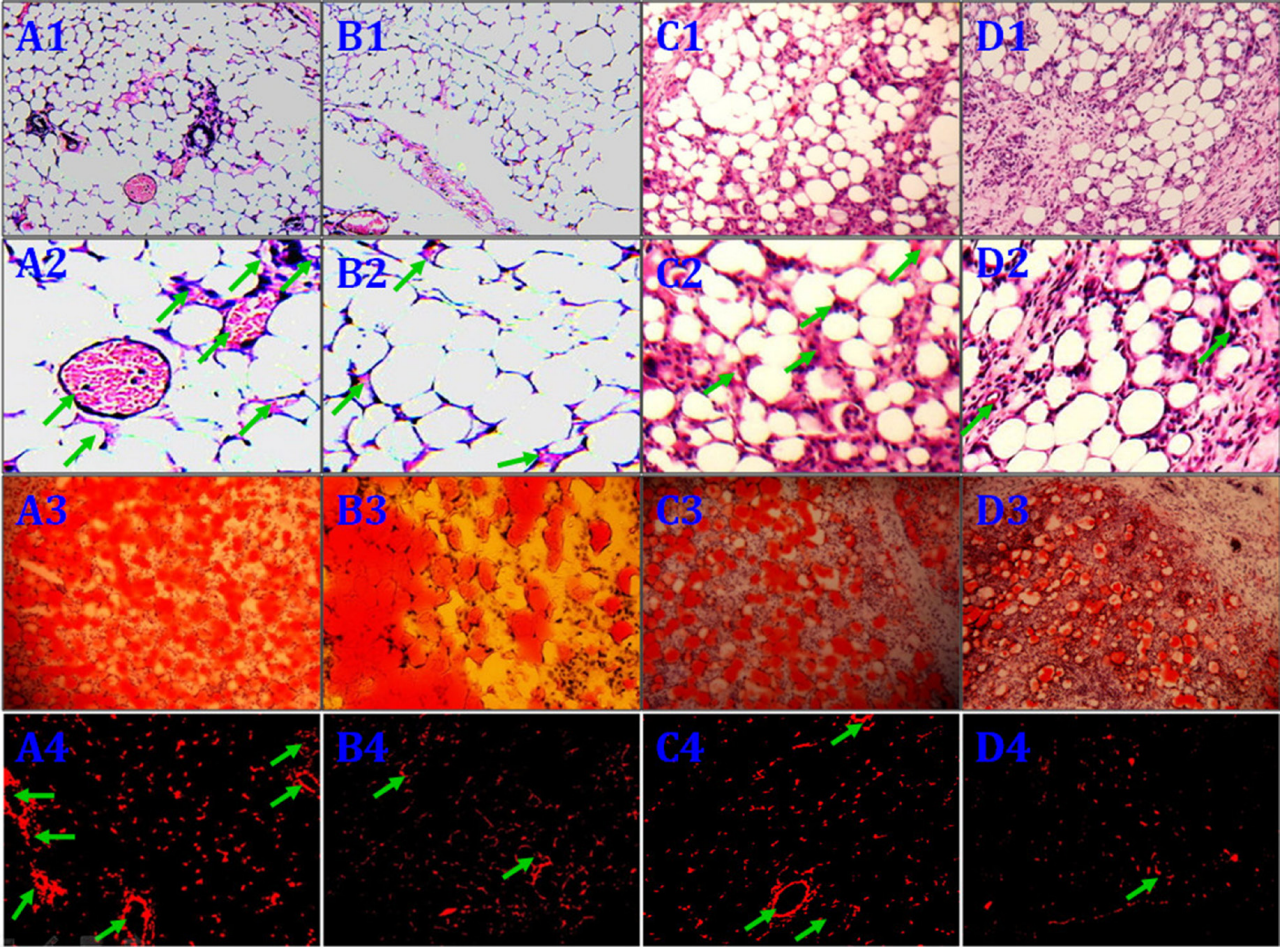
Histological evaluation of the neogenetic fat flaps after 12 weeks Group (**A**) flaps consisted predominantly of mature adipose tissue and showed less fibrosis than flaps in the other groups A1.–2. Group (**B**) flaps also consisted predominantly of mature adipose tissue and showed less fibrosis than group (**C** and **D**) flaps B1.– 2. Group C C1.– 2. and D D1.–2. flaps consisted partially of mature adipose tissue and showed more fibrosis than flaps in the other two groups. In contrast to cells in groups C and D, CD54^+^rASCs in group B contained more large oil red O-positive lipid droplets in their cytoplasm, and HIF-1α transfection (group A) augmented this effect A3.–D3. DiI^+^ cells were detected in neogenetic mature adipose tissue, indicating that these mature adipocytes differentiated from DiI-labeled rASCs A4.–D4. MVD: arrow. (A1.–D1., A3.–D3., A4.–D4. ×100, A2.–D2. ×400).

No animals died during the 12 weeks following implantation. Macroscopic findings indicated that regenerated tissue flaps had formed, and these were excised carefully for testing (Figure 5). Neogenetic fat flap wet weights were measured using an electronic balance (Table 2). Differences were found between groups A and B, groups A and C, groups A and D, groups B and C, groups B and D, and groups C and D (Figure 6). H&E staining results showed that regenerated tissue flaps in all groups were composed of mature adipose tissue. Compared with the other three groups, group A contained the most mature adipose tissue and showed no signs of fibrosis. Group B also consisted predominantly of mature adipose tissue and showed less fibrosis than groups C or D. Groups C and D partially consisted of mature adipose tissue and showed more fibrosis than groups A and B. Moreover, the collagen sponges were observed to have degraded over the course of 12 weeks after implantation *in vivo* (Figure 7).

**Table 2:** Wet weights in each group (mg, *n* = 20)

group	A	B	C	D
Wet weight	*532.6 ± 61.4	^*^417.8 ± 52.5	^#^226.3 ± 43.7	163.5 ± 38. 9

*P < 0.01, **P < 0.01, ^#^*P* < 0.01.

### Microvessel density (MVD) of regenerated fat flaps

**Figure 8:**
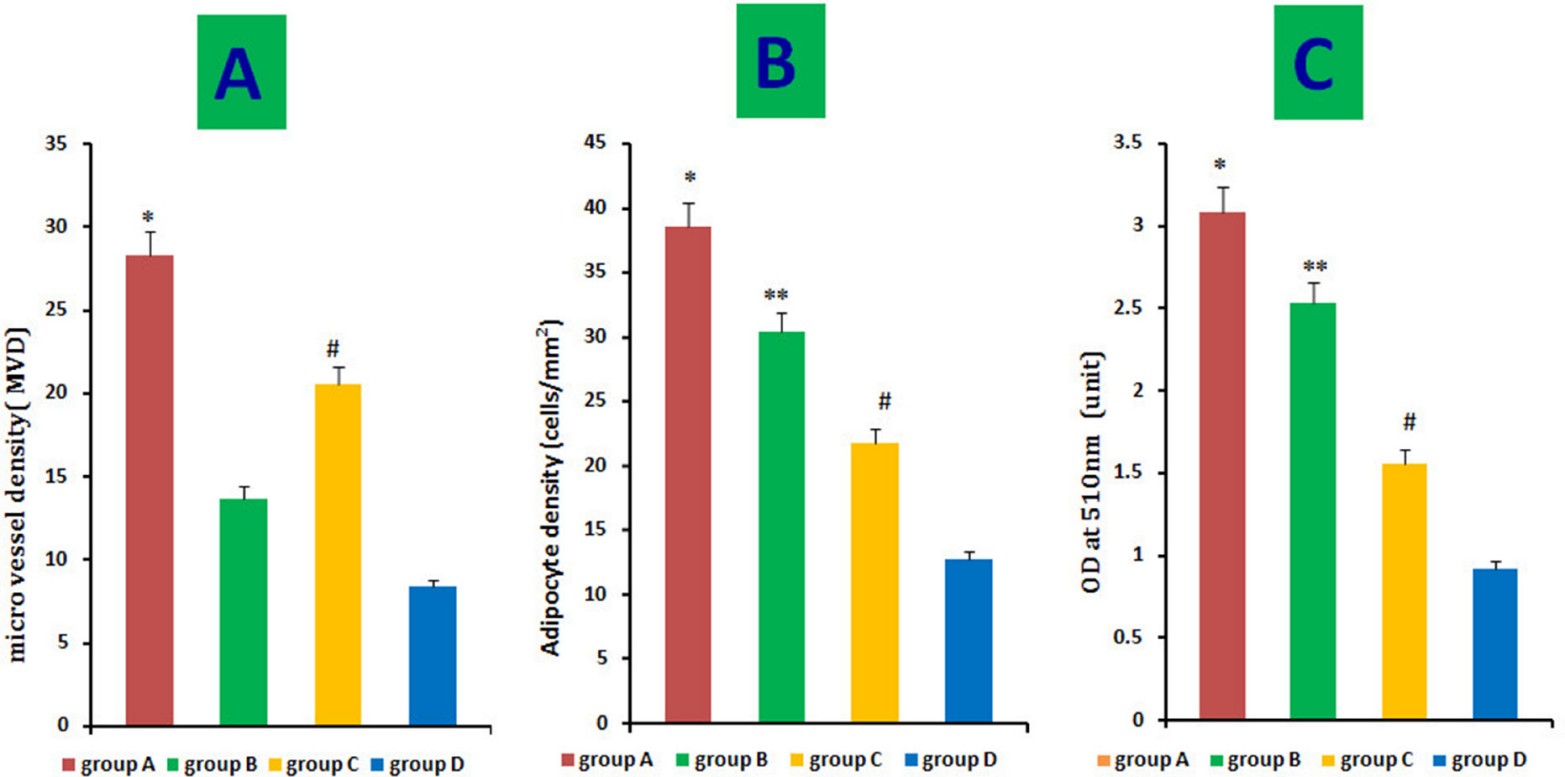
Quantitative measurement of adipogenesis and angiogenesis Adipocyte density and intracellular lipid content were higher in groups (**B**) and (**C**) than in the control group, and HIF-1α transfection (**A**) augmented this effect (B–C). Histological evaluation of 10 fields/section taken from the center of the mature adipose tissue showed that the MVD was much higher in group A than in the other three groups, and MVD in group C was much higher than that in group B and A, as determined by the *t-test* C. *P < 0.01, **P < 0.01, ^#^*P <* 0.01.

We histologically evaluated 10 fields/section from the centers of regenerated fat tissues to assess MVD. MVD was much higher in groups A than that of in the other three groups. In contrast, few microvessels were observed in group D. However, HIF-1α-transfected CD54^-^rASCs (group C) promoted neovascularization more so than untransfected CD54^-^rASCs (group D) or untransfected CD54^+^rASCs (group B). Thus, HIF-1α transfection enhanced revascularization. (Figures 7, 8).

### Oil red O staining and quantitative measurement of adipogenesis

Red-fluorescent cells were observed in regenerated fat flaps by fluorescence microscopy, indicating that these mature adipocytes had differentiated from DiI-labeled rASCs (Figure 7A4–7D4). Adipogenesis and lipid vacuole formation in neogenetic mature adipose tissue sections were assessed by oil red O staining. CD54^+^rASCs (group B) contained more large oil red O-positive lipid droplets in their cytoplasm, and transfection with HIF-1α (group A) augmented this effect. CD54^-^rASCs transfected with HIF-1α (group C) and CD54^-^rASCs contained fewer oil red O-positive lipid droplets (Figure 7). There were differences between groups A and B, groups A and C, groups A and D, groups B and C, groups B and D, and groups C and D in adipocyte density and lipid concentration (Figure 8).

### Paracrine-related genes and proteins expression in neogenitic fat flaps

**Figure 9:**
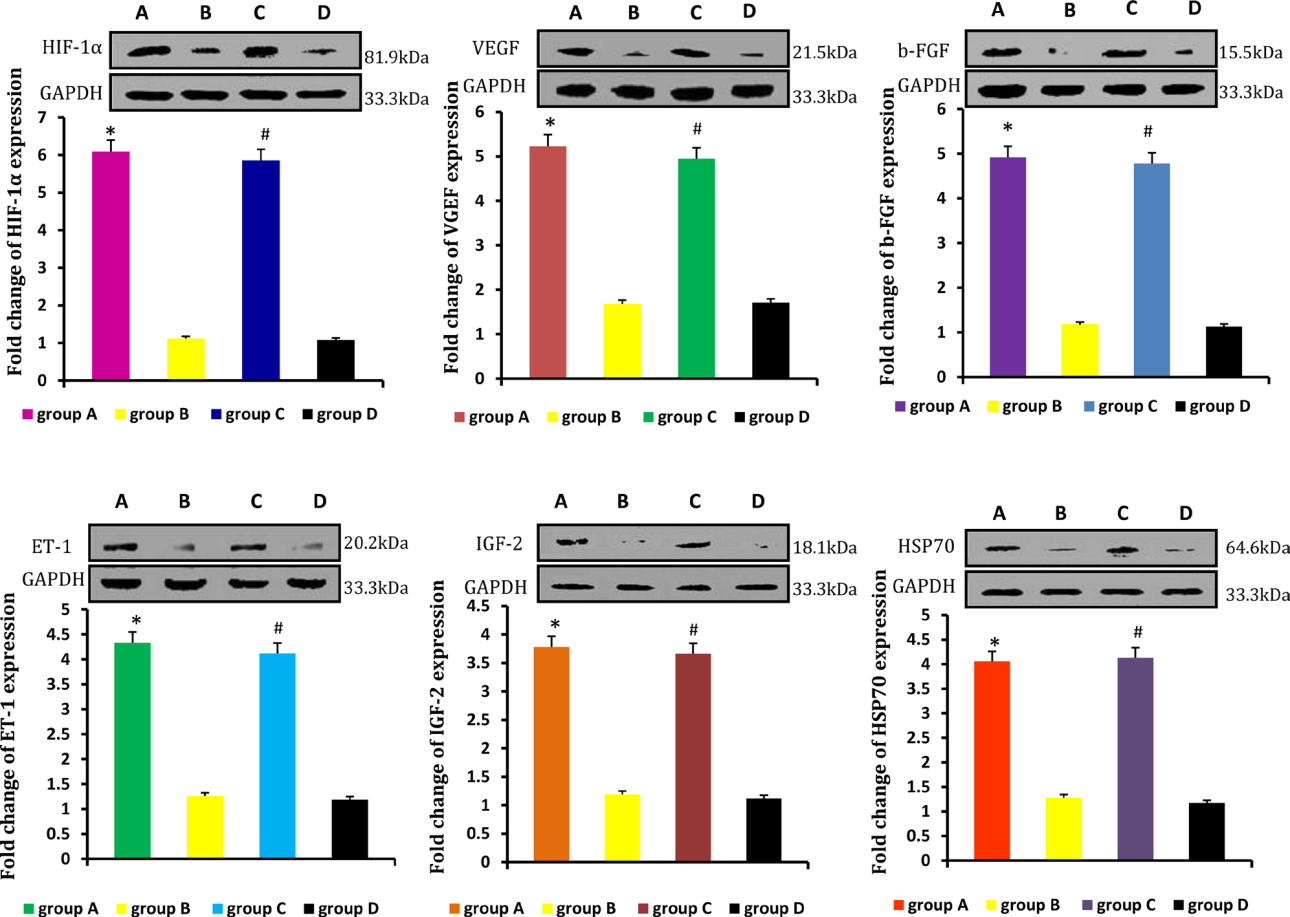
HIF-1α, VEGF, b-FGF, ET-1, IGF-2, and HSP70 mRNA levels were higher in the neogenetic fat flaps of groups A and C than in those of the other two groups *P < 0.01, ^#^*P* < 0.01.

**Figure 10:**
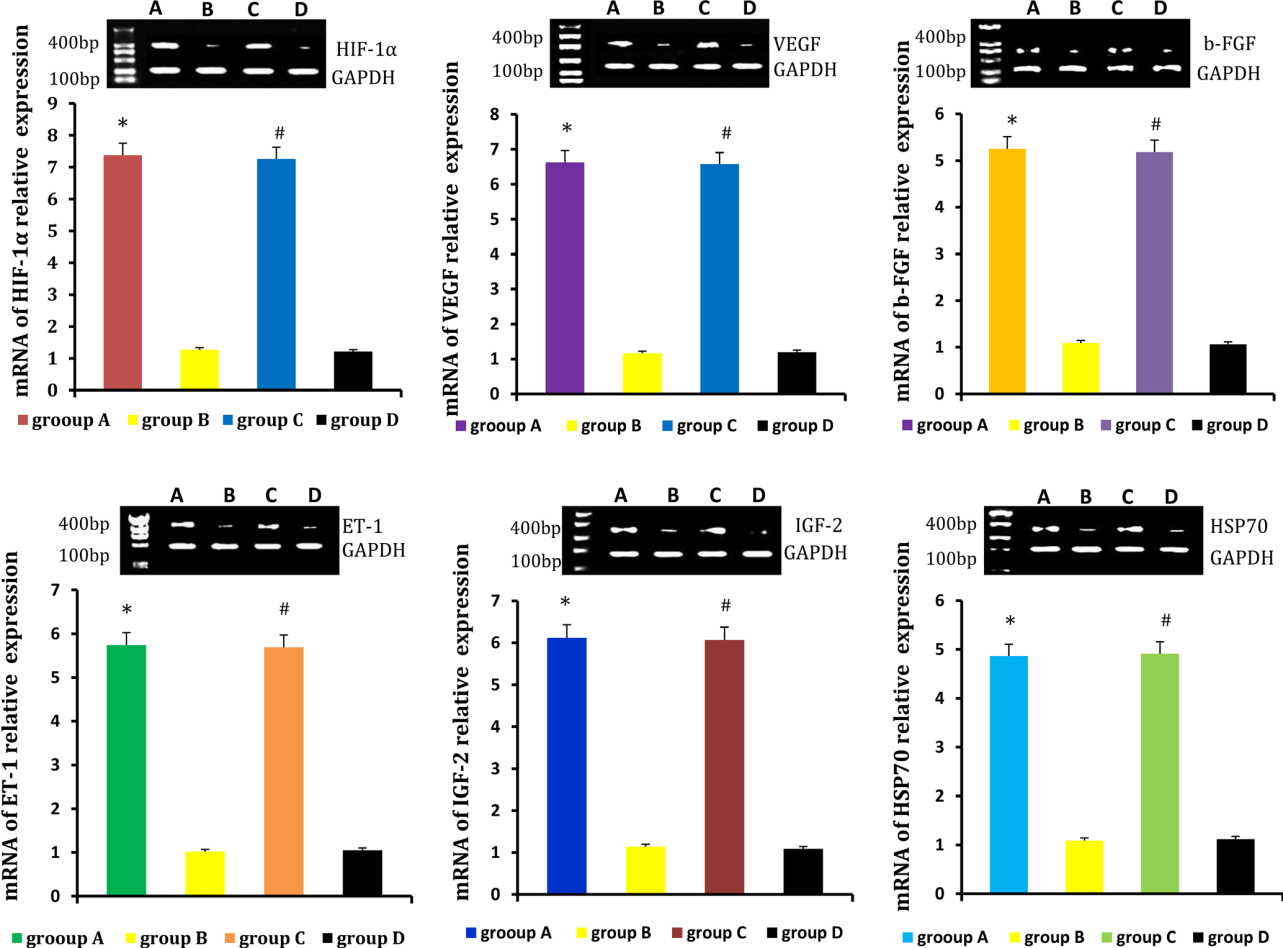
HIF-1α, VEGF, b-FGF, ET-1, IGF-2 and HSP70 protein levels were higher in the neogenetic fat flaps of groups A and C than in those of the other two groups *P < 0.01, ^#^*P* < 0.01.

Relative levels of HIF-1α, VEGF, b-FGF, ET-1, IGF-2, HSP70 were detected by real-time qPCR and western blot. Levels were higher in neogenetic fat flaps of groups A (HIF-1α-transfected CD54^+^rASCs) and C (HIF-1α-transfected CD54^-^rASCs) than in the other two groups (untransfected rASCs). (Figures 9, 10).

### Adipogenesis-related genes and proteins expression in neogenitic fat flaps

**Figure 11:**
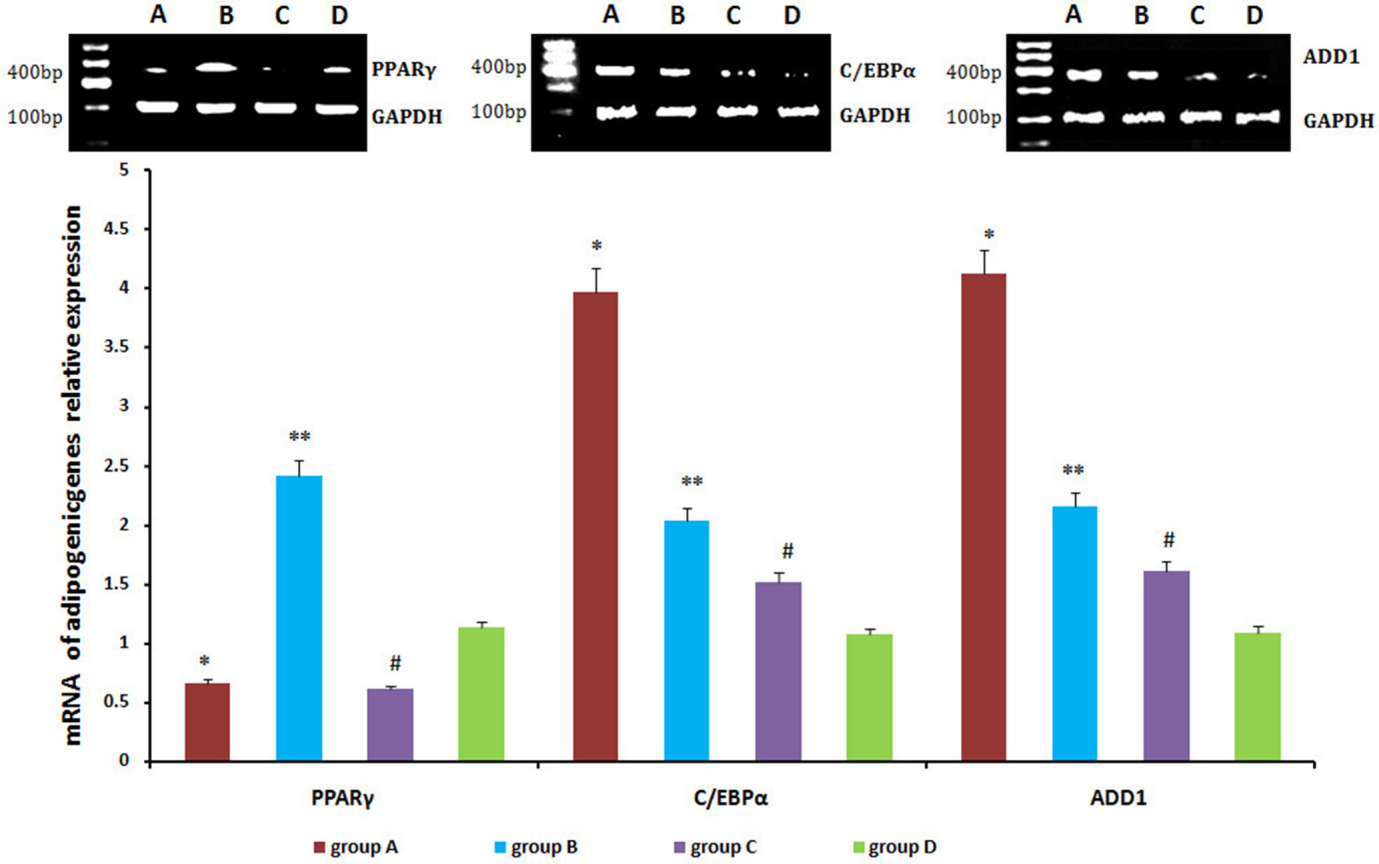
C/EBPα and ADD1 mRNA levels were higher in neogenetic fat flaps of group A than in the other three groups PPARγ was downregulated in the neogenetic fat flaps of groups A and C compared with the other two groups. *P < 0.01, **P < 0.01, ^#^*P <* 0.01.

**Figure 12:**
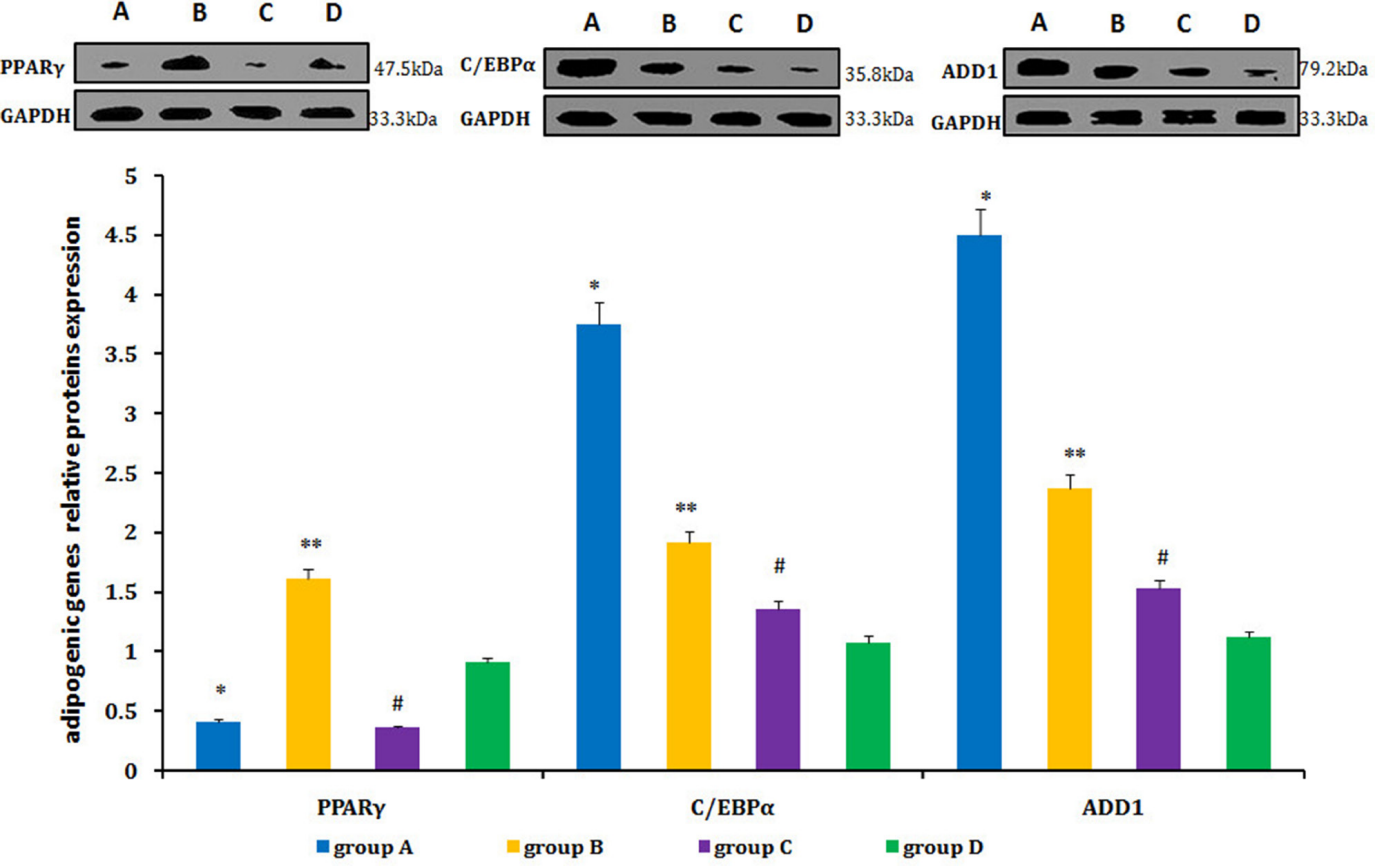
C/EBPα and ADD1 protein levels were higher in the neogenetic fat flaps of group A than in the other three groups PPARγ was downregulated in neogenetic fat flaps of groups A and C compared with the other two groups. *P < 0.01, **P < 0.01, ^#^*P <* 0.01.

Relative levels of PPARγ, C/EBPα, and ADD1 were detected by real-time qPCR and western blot. PPARγ expression was lower in neogenetic fat flaps of groups A (HIF-1α-transfected CD54^+^rASCs) and C (HIF-1α-transfected CD54^-^rASCs) than in the other two groups (untransfected rASCs). However, C/EBPα and ADD1 levels were increased in groups A and B compared with groups C and D (Figures 11, 12).

## DISCUSSION

Surgical reconstruction procedures using autologous fat flaps are effective treatment approaches for various soft tissue defects caused by congenital deformities, traumatic injuries, or tumor resection [22–24]. However, limitations in clinical practice include poor retention of autologous fat and the occurrence of secondary donor-site deformities. Engineering vascularized soft tissue flap grafts may provide clinical alternatives to autologous flap tissues.

HIF is a transcription factor induced under hypoxic conditions. HIF-1α migrates into the nucleus to form a stable heterodimer with HIF-1β or binds to histone acetylation enzymes to avoid degradation [25]. HIF-1α induces VEGF, platelet-derived growth factor (PDGF), and b-FGF expression, thereby enhancing angiogenesis and improving oxygenation of the environment [26–31]. However, the effects of HIF-1α in ASCs remained unclear. Some studies showed that HIF-1α is involved in cell proliferation during hypoxia [32–34].

This study assessed the adipogenic capacity of rASCs based on the CD54 marker, and found that rASCs with high CD54 expression exhibited stronger adipogenic differentiation potential *in vitro*. We examined rASCs proliferation and paracrine function with or without HIF-1α transfection *in vitro* and *in vivo*, and found that HGF, VEGF, ET-1, b-FGF, IGF-2, and HSP70 were upregulated in transfected rASCs. These results showed for the first time that rASCs modified by HIF-1α exhibit enhanced proliferation and paracrine function. Furthermore, macroscopic and histopathological findings suggested that CD54^+^rASCs exhibited greater adipogenic differentiation potential *in vivo* than CD54^-^rASCs, and transfecting CD54^+^rASCs with HIF-1α augmented this effect. HIF-1α gene modification or hypoxia preconditioning accelerated rASCs angiogenic potential, which was consistent with previous reports [33, 35].

PPARγ, C/EBPα, ADD1, and growth factors/cytokines are closely involved in adipogenic differentiation [19, 36–37]. PPARγ and C/EBPα are transcription factors in different signaling pathways, and ADD1 expression can be used to represent degree of adipogenic differentiation [38–39]. In our study, C/EBPα and ADD1 levels were increased, and PPARγ levels were decreased in HIF-1α-transfected CD54^+^rASCs.

The transcription factor nuclear receptor PPARγ is an essential and sufficient factor for inducing adipocyte differentiation, and serves as a key marker of the adipogenic switch. However, in our work, PPARγ expression was decreased with rASCs adipogenic differentiation, which was promoted by HIF-1α transfection. Other work showed that hypoxic conditions enhanced the adipogenic potential of MSCs and that HIF-1α overexpression induced lipid droplet accumulation, while PPARγ inhibition upregulated adipocyte-specific gene expression [40]. These results demonstrated that HIF-1α-induced adipogenic differentiation is independent of the PPARγ-regulated pathway. Thus, HIF-1α may induce rASCs adipogenic differentiation through a C/EBPα-dependent pathway. Based on our results, the PPARγ pathway may not be uniquely necessary for adipogenic differentiation.

**Figure 13:**
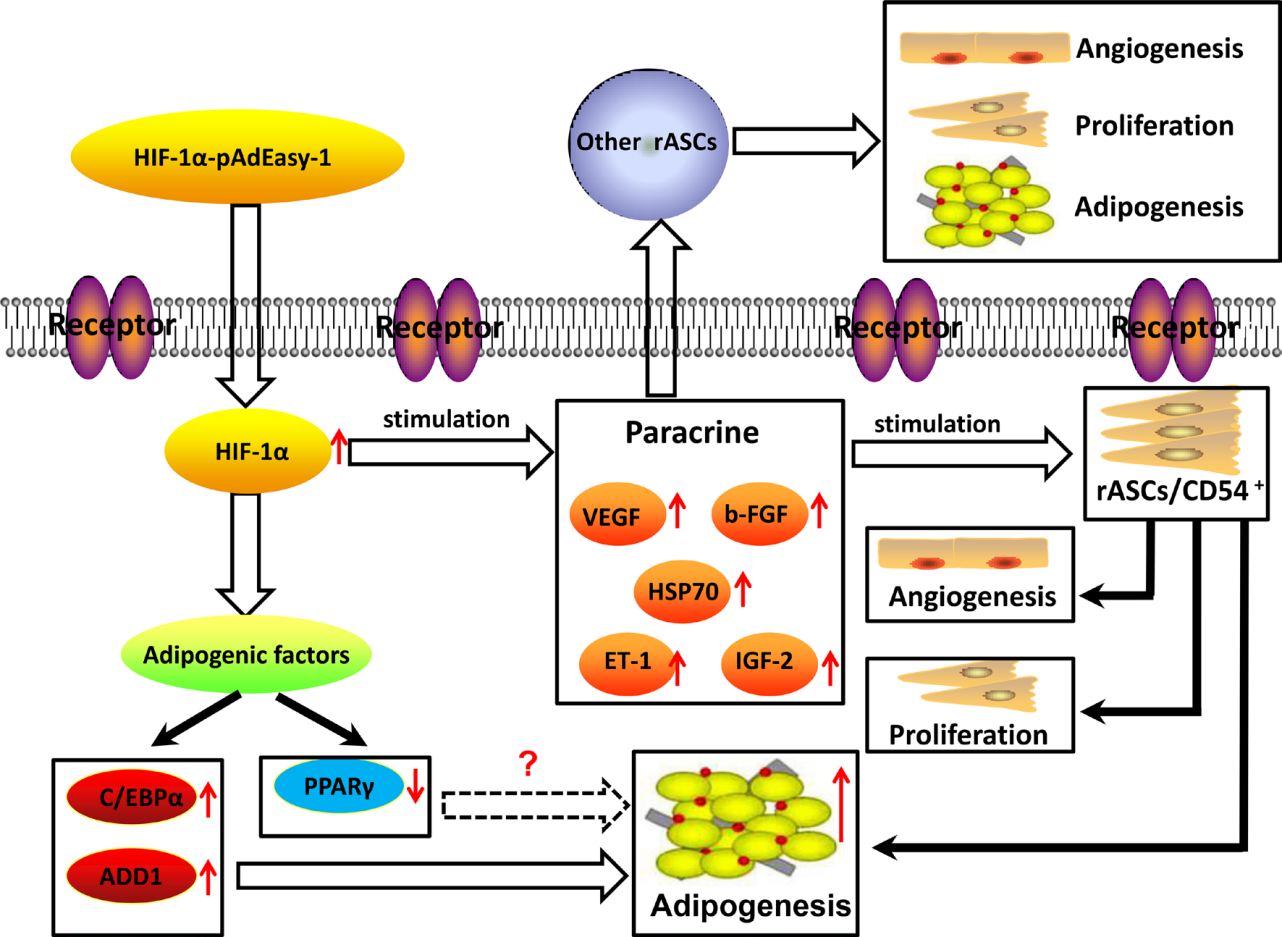
Graphical model of signaling in rASCs adipogenic differentiation CD54^+^rASCs exhibit stronger adipogenic differentiation potential *in vitro* and *in vivo*. HIF-1α transfection augmented this effect, and upregulated cell proliferation and paracrine function. rASCs adipogenic differentiation could thus be prompted by HIF-1α in a C/EBPα-dependent, rather than PPARγ-dependent pathway.

ASCs directly form new vascular networks by differentiating into endothelial cells [41], and paracrine functions are also involved, e.g., in the secretion of angiogenic factors, such as VEGF, ET-1, b-FGF, and PDGF [42–43]. Our results showed that paracrine function and MVD were both increased in fat flaps containing modified rASCs. Angiogenesis is not the only process affected by paracrine signals; previous studies demonstrated that paracrine signals from MSCs promote cell proliferation, migration, and secretion independently of promoting angiogenesis [44]. Moreover, growth factors are necessary for efficient differentiation of ASCs into adipocytes [36]. Since HIF-1α-transfected rASCs not only exhibited enhanced proliferation and paracrine function, but also facilitated vascularized fat flap regeneration, HIF-1α may promote early transplant neovascularization and enhanced rASCs adipogenic differentiation (Figure 13).

## MATERIALS AND METHODS

### rASCs isolation, expansion, and identification

Twenty subcutaneous adipose tissue samples were obtained from the groin fat pad of adult rabbits under anesthesia. Fibrous tissue was excised from fat pads and minced well with scissors at room temperature (RT). Experimental methods describing rASCs expansion, culture, and identification were described previously [45–46].

### DiI labeling, flow cytometric analysis, and immunoselection of rASCs

Passage-3 (P3) rASCs were collected and labeled with 1,1′-dioctadecyl-3,3,3′,3′-tetramethylin docarbocyanine perchlorate (DiI) for 24 h. *HIF-1α* was recombined with the target fragment, and the recombinant gene was introduced into a pAdEasy-1 adenoviral vector system. The virus was packaged, and titer was assessed. rASCs red autofluorescence was analyzed by inverted fluorescence microscopy and imaging (Leica, Germany). Flow cytometry was performed using P3 rASCs. Cells were trypsinized using Accutase (Innovative Cell Technologies, San Diego, CA, USA), centrifuged at 1500 rpm for 10 min and washed with Dulbecco's phosphate-buffered saline (PBS) containing 0.5% bovine serum albumin (Sigma). Cells were filtered through a 70-mm nylon membrane, and cells were counted using a hemocytometer. 1×10^6^ cells were incubated with either fluorescein isothiocyanate (FITC)- or phycoerythrin (PE)-conjugated antibodies for 30 min. The following surface CD markers were tested: CD29/PE, CD31/PE, CD34/FITC, CD44/FITC, CD45/FITC, CD54/FITC, CD73/FITC, CD90/FITC, CD105/PE, HLA-ABC/FITC, and HLA-DR-DP-DQ/FITC (BD Biosciences, NJ, USA). Ten thousand events were acquired for each CD surface marker using a Becton Dickinson FACSCalibur flow cytometer. Data were analyzed using CellQuestPro acquisition software (BD Bioscience, Franklin Lakes, NJ, USA). CD54 surface antigen-positive (CD54^+^) cells were immunoselected from the heterogeneous mix of rASCs using immunomagnetic beads from a MACS^®^ microbead kit (Miltenyi Biotech) according to the manufacturer's protocol. CD54^+^ and CD54^-^ rASCs were centrifuged, resuspended in growth medium, and seeded in cell culture flasks for continued expansion.

### HIF-1α-transfected rASCs

*HIF-1α* was introduced into a pAdEasy-1 adenoviral vector system. The virus was packaged, and titer was assessed. P5 CD54^+^ and CD54^-^ rASCs were infected with the HIF-1α-encoding adenovirus at 90% confluence, as determined by fluorescence microscopy. Prior to infection, cells were washed with infection buffer three times and incubated at RT for 10 min. Infection was carried out with a multiplicity of infection of 50 pfu/cell in 1 ml of infection buffer at RT for 60 min. After infection, rASCs were incubated at 37°C with 5% CO_2_ in a humidified atmosphere. Three d later, rASCs were subcultured for use in the following experiments.

### Influence of HIF-1α on rASCs proliferation *in vitro*

For cell proliferation assays, HIF-1α-transfected rASCs, blank-transfected rASCs, and control rASCs were cultured in 96-well plates at 2 × 10^4^ cells/well with culture medium for up to 9 d. rASCs proliferation was measured using Cell Counting Kit 8 (CCK-8, Kumamoto, Japan) assay and a microplate reader (ELx800, BioTek) at 450 nm, as described previously [6].

### Paracrine function of rASCs after transfected HIF-1α *in vitro*

After 3 and 7 d, untransfected and transfected rASCs culture supernatants were collected for analysis by ELISA. HGF, VEGF, b-FGF, ET-1, IGF-2 and HSP70 levels were measured using Quantikine colorimetric sandwich ELISA kits (R&D Systems, Minneapolis, MN, USA) according to the manufacturer's instructions.

### Influence of HIF-1α on rASCs adipogenic differentiation *in vitro*

For adipogenesis assays, HIF-1α-transfected CD54^+^rASCs (group A), CD54^+^rASCs (group B), HIF-1α-transfected CD54^-^rASCs (group C), and CD54-rASCs(group D) were cultured in 6-well plates with adipogenic induction medium containing 200 μM indomethacin, 10 μM insulin, 0.5 mM 3-isobutyl-1-methylxanthine, and 1 μM dexamethasone at a seeding density of 500 cells/mm^2^ for up to 14 d. Adipogenic differentiation was determined by oil red O staining. Briefly, cultured cells were air dried for 30 min, and then fixed in 10% formalin for 5 min. Cells were rinsed three times with distilled water, and immersed in absolute propylene glycol for 5 min to avoid transferring water into the oil red O. Cells were stained in oil red O solution for 8 min at 60°C in an oven, rinsed with 85% propylene glycol for 5 min,. rinsed with distilled water, and then stained in Gill's hematoxylin solution for 30 sec. Cells were thoroughly washed with running water for 3 min, rinsed twice with distilled water, and mounted with glycerin jelly or aqueous mounting medium. Upon observation of the samples, lipids appeared red and nuclei appeared pale blue. Adipocyte density for each sample was measured in 10 visual fields under the same magnification in a double-blind manner. Cell numbers were normalized to square millimeters. Lipids were extracted from cells with 100% isopropanol and gentle shaking for 5 min. Lipid concentrations were measured based on absorbance at 510 nm in triplicate for each sample.

### rASCS-loaded scaffold implantation *in vivo*

All animal procedures were performed in accordance with the guidelines of the Guangxi Medical University Animal Care and Use Committee. Twenty New Zealand rabbits (aged 3–4 months old, weighing 2.0 ± 0.3 kg) were used to establish an autologous cell implantation model. CD54^+^ and CD54^-^ rASCs were cultured with adipogenic induction media for one week before implantation. Collagen scaffolds loaded with cell suspensions were essentially gel masses containing 1.0 ml of 1 × 10^7^/ml HIF-1α-transfected CD54^+^rASCs (group A), 1.0 ml of 1 × 10^7^/ml CD54^+^rASCs (group B), 1.0 ml of 1 × 10^7^/ml HIF-1α-transfected CD54^-^rASCs (group C), or 1.0 ml of 1 × 10^7^/ml CD54^-^rASCs (group D). All compounds were implanted into the muscular fasciae on the backs of rabbits. Each implantation site randomly received one of these four scaffolds.

### Neogenetic fat flap wet weight measurement and histopathological examination

At 12 weeks post-implantation, all transplants (i.e., neogenetic fat flaps) were excised from the muscular fasciae and weighed using a standard electronic balance. Transplants were then fixed in formalin and embedded in paraffin. Tissue sections from the center of the dissected regenerative tissue biopsy were stained with hematoxylin and eosin (H&E) using standard procedures and examined by light microscopy. MVD was assessed by counting microvessels in 10 fields of each H&E-stained slide (40× magnification; performed by two blinded reviewers).

### Oil red O staining and quantitative measurement of adipogenesis *in vivo*

Neogenetic fat flap tissues samples were embedded in paraffin, cut into 5-μm-thick slices, and observed by fluorescence microscopy. Cyrosections were stained with oil red O and washed twice with PBS. Adipocyte densities were measured in a double-blind manner in 10 visual fields under the same magnification. Cell numbers were normalized to square millimeters. Lipids were extracted from cells with 100% isopropanol and gentle shaking for 5 min. Lipid concentrations were measured based on absorbance at 510 nm in triplicate for each sample.

### Real-time quantitative PCR (qPCR)

Total RNA was extracted from each group using TRIzol according to the manufacturer's protocol (Invitrogen, USA). First-strand cDNA was synthesized from 1 μg of RNA with viral reverse transcriptase (TaKaRa, Japan) and used for real-time qPCR. HIF-1α, VEGF, b-FGF, ET-1, IGF-2, HSP70, PPARγ, C/EBPα, and ADD1 levels were quantified using an ABI 7300 real-time PCR system (Applied Biosystems, USA) with SYBR green PCR reaction mix (TaKaRa, Japan). Primers are listed in Table 3. The following program was used: 95°C for 5 min, 40 cycles of 95°C for 15 sec, annealing for 1 min, and 72°C for 30 sec. A melting analysis and agarose gel electrophoresis were performed to confirm PCR product specificities. Relative gene expression was analyzed using the 2^–ΔΔCt^ method, normalized to GAPDH expression, and data are presented as fold change relative to the control.

**Table 3:** Primer sequences

Gene name (rabbit)	Forward (5′ to 3′)	Reverse (5′ to 3′)
HIF-1α	TGAAACTCAAGCAACGGTCA	GGCTCCTTCTTCAGTTTGTCA
VEGF	CTCTACCTCCACCATGCCAA	CTCCAGGCTTTCATCATTGCAG
b-FGF	TTCACAGCCCTGACCGAGA	CTGTCCCTTGTGCCGTCCA
ET-1	CCAAGCAGGAACGGAACTCA	CACTTTCTGCTCTCGGTGGA
IGF-2	CATTGTGGAGGAATGCTGCTTC	CCCGGTGTCAAGCTGAAATAAC
HSP70	AACAACCTGCTGGGGCGCTT	CCTTGCCCGTGCTCTTGTCGGT
PPARγ	AATCAAAGTGGAACCTGCATC	TTCGGAAGAAACCCTTGCAT
C/EBPα	AGCCGATATCTTGTATCTAGCCT	CTCATTTTGGCAAGTATCTGAGC
ADD1	TATGACCGCAAACGTCCCG	ATGAGCTGAGACCACCCG
GAPDH	AATCCACTGGCGTCTTCACC	GCCCCTCCACAATGCCGA

### Protein extraction and western blotting

Neogenetic fat flap samples from each group were harvested for western blot analysis, and whole cell extracts were obtained. Cell pellets were sonicated in extraction buffer, and extracts were quantified using a Bio-Rad DC protein assay kit (Bio-Rad, Hercules, California, USA). Equal amounts of protein were resolved by 4–12% SDS-PAGE and transferred to PVDF membranes (Millipore, Bedford, Massachusetts, USA). Membranes were blocked with blocking solution (Pierce, Rockford, Illinois, USA). The following primary antibodies (all anti-rabbit) were used: HIF-1α, VEGF, b-FGF, ET-1, IGF-2, HSP70, PPARγ, C/EBPα, and ADD1 (all from Abcam, London, UK). Horseradish peroxidase-conjugated secondary antibodies and enhanced chemiluminescence substrates (Supersignal West Dura Detection System; Pierce) were used for primary antibody detection.

### Statistical analysis

Data are shown as means ± standard deviations (SD). We used analysis of variance (ANOVA) to compare group means. This approach accounts for intra- and inter-group variation. When ANOVA of the four means revealed statistically significant differences, the means of any two groups were compared using paired *t*-tests. *P <* 0.05 (two-tailed) was considered statistically significant. All data were analyzed using SPSS for Windows version 17.0 (Chicago, Illinois, USA).
